# Supplementary figures and images for: Pneumothorax after computed tomography-guided lung biopsy: Utility of immediate post-procedure computed tomography and one-hour delayed chest radiography
Source: PLoS One. 2023 Apr 19;18(4):e0284145. doi: 10.1371/journal.pone.0284145 (PMC10115279; doi:10.1371/journal.pone.0284145)

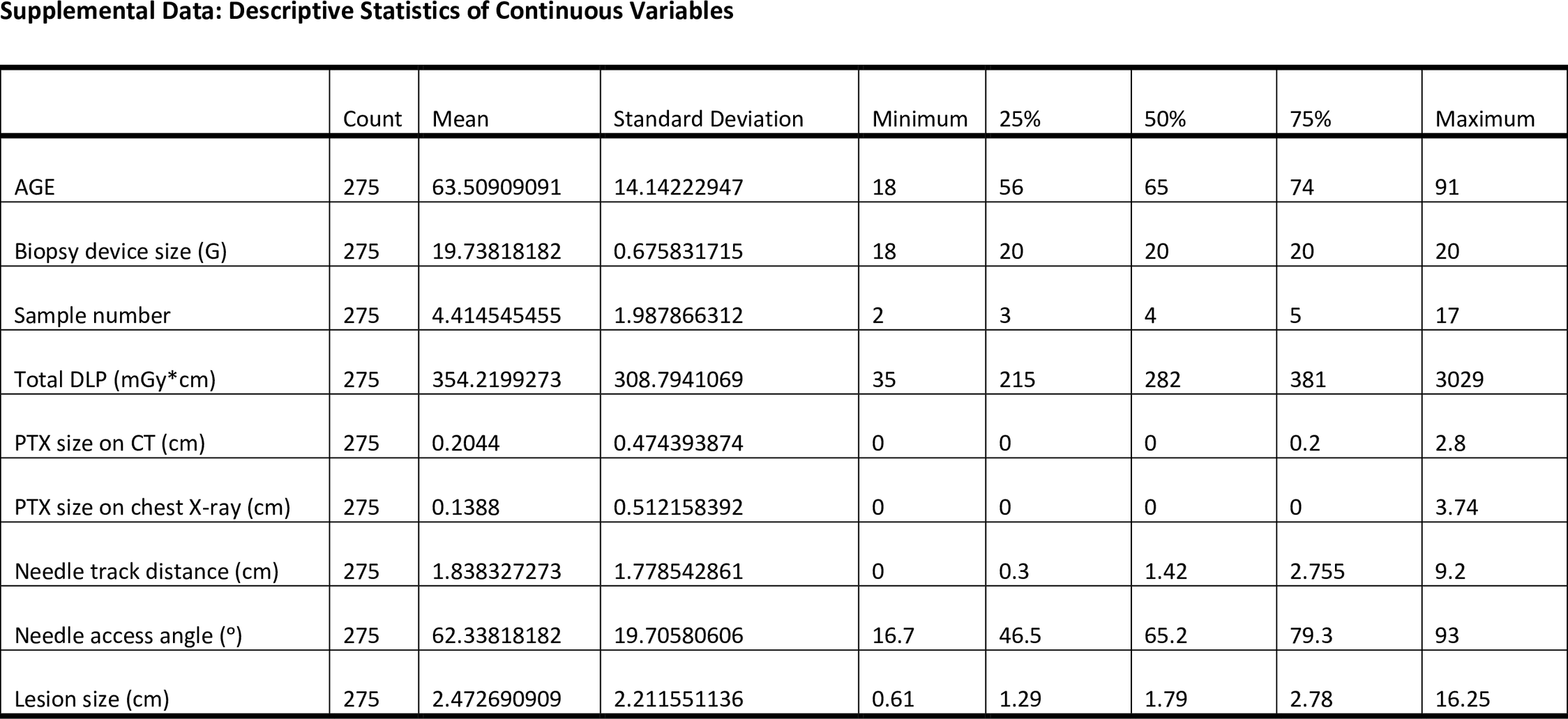

Supplement: S1 Table — (TIF) [file pone.0284145.s002.tif]
