# Supplementary material for: Pneumothorax after computed tomography-guided lung biopsy: Utility of immediate post-procedure computed tomography and one-hour delayed chest radiography
Source: PLoS One. 2023 Apr 19;18(4):e0284145. doi: 10.1371/journal.pone.0284145 (PMC10115279; doi:10.1371/journal.pone.0284145)
Supplement: Attachment — Submitted filename: Response to Reviewers.docx [file pone.0284145.s003.docx]

PLOS ONE

Manuscript number: PONE-D-22-29210

Response to Reviewers

Dear Editor-in-Chief:

We appreciate the time and effort you and the reviewers dedicated to providing feedback. We have revised the manuscript according to the reviewers’ suggestions, and the changes were properly highlighted. Also, supporting supplemental information was provided. Please see the below list for the response to the suggestions.

1. Added a dedicated “ethics statement” subheading in materials and methods that contains additional requested details about the institutional IRB involved in our research.
2. Re-formatted the entirety of the references section to comply with ICMJE guidance.
3. Added a “supporting information” section with raw data and additional statistical analysis of our research and uploaded a corresponding file with raw data and a table with supplemental statistical analysis.
4. An email has been sent requesting the removal of the previously requested direct billing option.

Several small, stylistic changes were made to the text to improve clarity/readability.

I appreciate your consideration, and I look forward to hearing from you.

Sincerely,

Jared Weinand
